# q-Space Imaging Yields a Higher Effect Gradient to Assess Cellularity than Conventional Diffusion-weighted Imaging Methods at 3.0 T: A Pilot Study with Freshly Excised Whole-Breast Tumors

**DOI:** 10.1148/rycan.2019190008

**Published:** 2019-09-27

**Authors:** Nicholas Senn, Yazan Masannat, Ehab Husain, Bernard Siow, Steven D. Heys, Jiabao He

**Affiliations:** From the Institute of Medical Sciences, School of Medicine, University of Aberdeen, Aberdeen AB25 2ZD, Scotland (N.S., S.D.H., J.H.); Breast Unit (Y.M., S.D.H.) and Department of Pathology (E.H.), Aberdeen Royal Infirmary, Aberdeen, Scotland; and MRI Unit, The Francis Crick Institute, London, England (B.S.).

## Abstract

**Purpose:**

To determine whether q-space imaging (QSI), an advanced diffusion-weighted MRI method, provides a higher effect gradient to assess tumor cellularity than existing diffusion imaging methods, and fidelity to cellularity obtained from histologic analysis.

**Materials and Methods:**

In this prospective study, diffusion-weighted images were acquired from 20 whole-breast tumors freshly excised from participants (age range, 35–78 years) by using a clinical 3.0-T MRI unit. Median and skewness values were extracted from the histogram distributions obtained from QSI, monoexponential model, diffusion kurtosis imaging (DKI), and stretched exponential model (SEM). The skewness from QSI and other diffusion models was compared by using paired *t* tests and relative effect gradient obtained from correlating skewness values.

**Results:**

The skewness obtained from QSI (mean, 1.34 ± 0.77 [standard deviation]) was significantly higher than the skewness from monoexponential fitting approach (mean, 1.09 ± 0.67; *P* = .015), SEM (mean, 1.07 ± 0.70; *P* = .014), and DKI (mean, 0.97 ± 0.63; *P* = .004). QSI yielded a higher effect gradient in skewness (percentage increase) compared with monoexponential fitting approach (0.26 of 0.74; 35.1%), SEM (0.26 of 0.74; 35.1%), and DKI (0.37 of 0.63; 58.7%). The skewness and median from QSI were significantly correlated with the skewness (ρ = −0.468; *P* = .038) and median (ρ = −0.513; *P* = .021) of cellularity from histologic analysis.

**Conclusion:**

QSI yields a higher effect gradient in assessing breast tumor cellularity than existing diffusion methods, and fidelity to underlying histologic structure.

**Keywords:** Breast, MR-Diffusion Weighted Imaging, MR-Imaging, Pathology, Tissue Characterization, Tumor Response

*Online supplemental material is available for this article.*

Published under a CC BY 4.0 license.

**Summary**

q-Space imaging yields a higher effect gradient to assess cellularity in breast cancer compared with conventional diffusion-weighted imaging methods by using a clinical 3.0-T MRI unit to image whole freshly excised breast tumors.

**Key Points**

■ The degree of tumor skewness obtained from q-space imaging (1.34 ± 0.77) was significantly higher than that obtained from existing diffusion imaging methods, and yielded a higher relative effect gradient.
■ The median and skewness from q-space imaging were significantly correlated with the median (ρ = −0.513, *P* = .021) and skewness (ρ = −0.468, *P* = .038) of cellularity from histologic analysis.

## Introduction

Breast cancer is the most prevalent cancer in women (1), with neoadjuvant chemotherapy treatment used before surgery to downstage locally advanced breast tumors and facilitate conservation surgery (2,3). Although a complete pathologic response can be achieved for up to approximately 60% of patients with triple-negative and human epidermal growth receptor 2–positive breast cancers (4,5), a significant proportion of patients progress or show no response to treatment, leading to unnecessary exposure to drug toxicity and delay to surgical intervention. Patients who positively respond to neoadjuvant chemotherapy treatment show a reduction in tumor cellularity (the percentage of tissue composed of tumor cells), determined as a reduction in the proportion of viable tumor tissue in posttreatment histologic analysis (6,7).

Diffusion-weighted imaging (DWI) is a conventional radiologic method that provides noninvasive assessment of cellularity by examining the extent that water self-diffusion is confined (8,9), with the proportion of viable tumor cellularity across whole tumors inferred as the amount of skewness present in histogram distributions (10). However, DWI has limited clinical application because of a low measurement-effect gradient and in turn sensitivity to treatment effectiveness (11–13). Whereas the monoexponential fitting approach reduces the susceptibility of DWI to acquisition configuration (14), the diffusion kurtosis imaging (DKI) (15) and stretched exponential model (SEM) (16) fitting approaches account for the complexity of diffusion in tissue, providing a more accurate representation of diffusion in breast carcinoma (17). However, the diffusion measurements obtained from these approaches come from idealistic models of diffusion and are unspecific to underlying tissue features (18).

q-Space imaging (QSI) eliminates the constraints introduced by modeling approaches, with measurements made directly from the diffusion pattern providing microstructure quantification in the brain (19,20) and sensitive assessment of the changes that arise from malignant transformation (21). QSI has been shown to be feasible on clinical MRI units because of recent hardware advances meeting the demand on magnetic field gradient, with application demonstrated in brain malignancies (22). We therefore hypothesized that measurements of breast tumor cellularity obtained with QSI have a higher effect gradient compared with other existing DWI techniques, and fidelity to the cellularity obtained from histologic analysis.

## Materials and Methods

**Figure 1:**
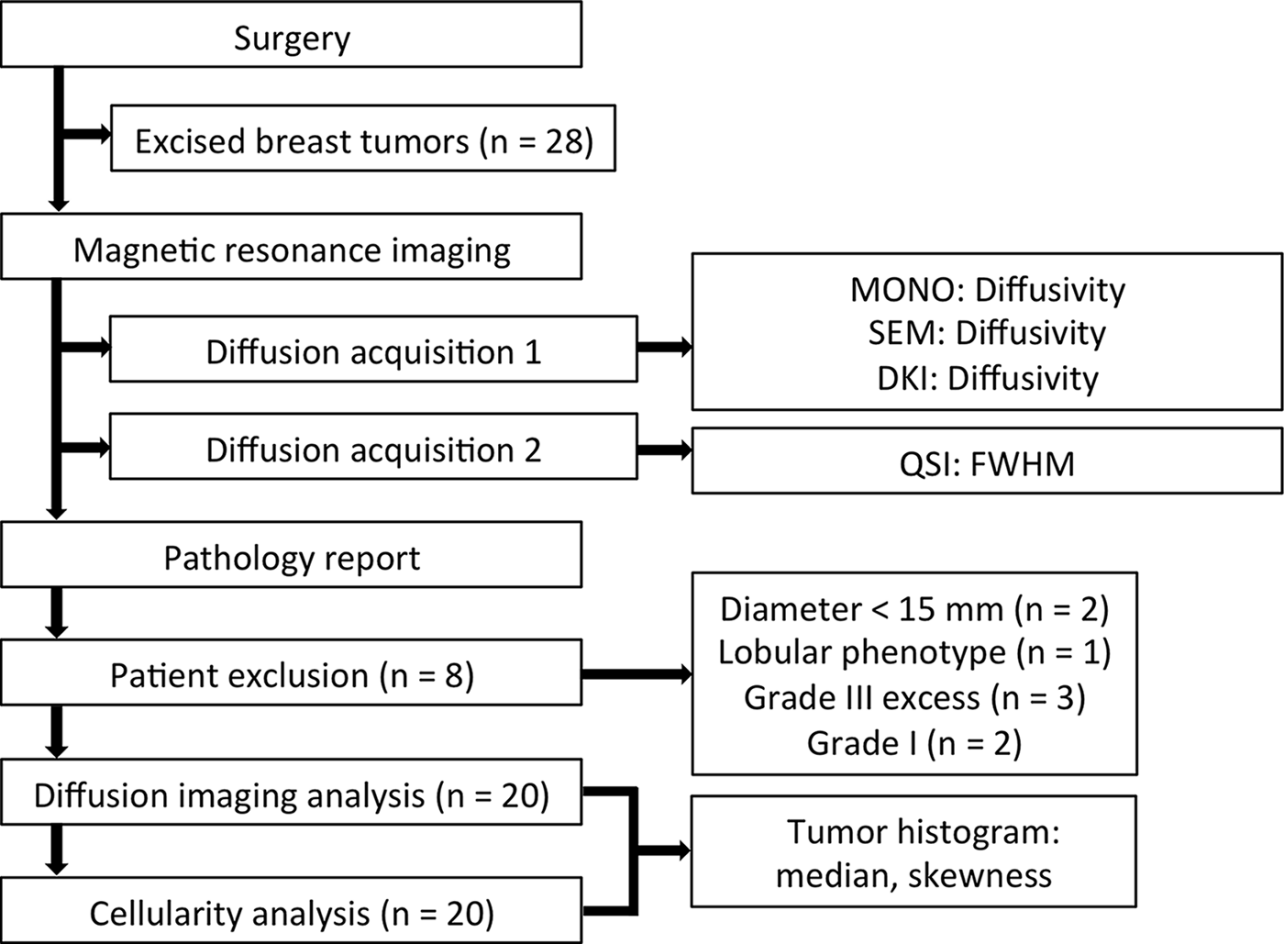
Study design. The study design adopted for evaluating q-space imaging (QSI) versus other diffusion-weighted imaging (DWI) techniques for the assessment of tumor cellularity in breast cancer application is shown. Diffusion acquisition 1 was analyzed by using fitting methods (monoexponential model [MONO], stretched exponential model [SEM], and diffusion kurtosis imaging [DKI]) at DWI to obtain respective measurements of diffusivity. Diffusion acquisition 2 was analyzed by using QSI to obtain the full width half maximum (FWHM) of the probability density function. Of the 28 tumors imaged in this study, eight were excluded due to the exclusion criteria of diameter < 15 mm, lobular phenotype, and tumor grading criteria, as reported in the final pathology. Tumor skewness was quantified from the histogram distributions obtained from the DWI methods and cellularity.

To test the study hypothesis, we conducted a prospective study in participants with breast cancer on a clinical MRI unit by using a series of DWI examinations performed on whole tumors freshly excised from participants. Participants were enrolled consecutively from Aberdeen Royal Infirmary (Aberdeen, Scotland) between August 2016 and June 2017 (Fig 1). The study was approved by the North West–Greater Manchester East Research Ethics Committee (identifier: 16/NW/0221) and signed written informed consent was obtained from the participants prior to entry into the study. Authors had control of data and information submitted for publication. Philips Healthcare (Best, the Netherlands) is acknowledged in this study for providing clinical scientist support.

### Participant Eligibility

Participants with grade II or III invasive carcinoma, tumor diameter greater than 15 mm, undergoing breast conservation surgery, with no chemotherapy or radiation therapy treatment before surgery were eligible. Among the 32 participants approached, all participants consented to the study and 28 specimens were imaged because of scanner availability. Twenty female participants (mean age, 57 years; age range, 35–78 years; 10 participants with grade II and 10 participants with grade III carcinoma) met the inclusion criteria on the basis of final histologic analysis (Table 1).

**Table 1:**
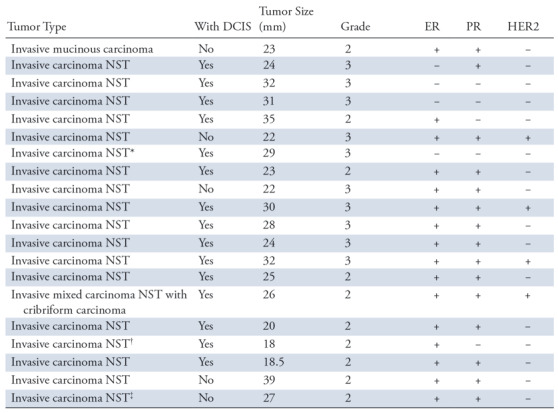
Tumor Characteristics

Note.—The tumor characteristics of each invasive carcinoma breast tumor are shown. Invasive lobular carcinoma types were not included in the study. *With DCIS* refers to the presence of ductal carcinoma in situ. Tumor size refers to the maximum diameter of tumor. There were three invasive carcinoma of no special type that showed less than 50% of special type tumor morphologic structure. + = positive, − = negative, DCIS = ductal carcinoma in situ, ER = estrogen receptor, HER2 = human epidermal growth factor receptor 2, NST = no special type, PR = progesterone receptor.

* Carcinoma with apocrine features.

^†^ Carcinoma with lobular features.

^‡^ Carcinoma with cribriform features.

### Tumor Inclusion

The excised whole tumor was placed in a sealed container filled with 10% formalin solution and immobilized by using a specially designed holding harness before overnight imaging for no delay to routine reporting. After histopathologic examination of the excised tumor, tumors with final diagnosis of lobular phenotype, tumor diameter less than 15 mm at histologic analysis, tumor downgraded to grade I, and tumors in excess of the grade III recruitment allocation were excluded (Fig 1).

### Image Acquisition

Images were acquired on a clinical 3.0-T MRI unit with maximum gradient strength of 80 mT/m (Achieva Tx; Philips Healthcare) by using a body coil for uniform transmission and a 32-channel receiver head coil for high sensitivity signal detection. All imaging volumes were centered on the tumor with sections on the horizontal plane and circular saturation bands positioned around the tumor to suppress the signal from formalin. Anatomic images were acquired by using a standard T1-weighted three-dimensional sequence (23) as follows: repetition time (TR) msec/echo time (TE) msec, 5.7/2.9; field of view, 141 × 141 mm^2^; 256 × 256 matrix; 28 sections; section thickness, 1.1 mm; and parallel acquisition acceleration factor, 1.5. Diffusion examinations were performed by using a multishot pulsed gradient spin-echo sequence (23) as follows: field of view, 141 × 141 mm^2^; section thickness, 2.2 mm; 64 × 64 matrix; in-plane resolution, 2.2 × 2.2 mm^2^; and seven to 10 sections depending on tumor size. Conventional DWI acquisition (for tumor delineation) was performed over two diffusion-weighted sequences (ie, *b* values) of 0 and 800 sec/mm^2^, as follows: diffusion time (δ/Δ), 15.3/27.5 msec; TR/TE, 3000/70 msec; one signal average; and duration, 2:21 minutes. The first diffusion imaging acquisition, or diffusion acquisition 1 (for monoexponential fitting approach, DKI, and SEM assessment), was performed over 17 linearly spaced *b* values from 0 to 2400 sec/mm^2^, as follows: spacing, 150 sec/mm^2^; δ/Δ, 18.7/31.5 msec; TR/TE, 3100/82 msec; two signal averages; and duration, 25:28 minutes. The second diffusion imaging acquisition, or diffusion acquisition 2 (for QSI assessment), was performed over 32 equidistant q values from 10.4 to 655 cm^−1^, equivalent to a maximum *b* value of 5000 sec/mm^2^, as follows: δ/Δ, 24.9/37.8 msec; TR/TE, 5900/94 msec; one signal average; and duration, 47:59 minutes.

### Image Analysis

To remove directionality, images of a specific diffusion weighting were computed as the voxel-wise average of images from three orthogonal diffusion directions of the corresponding diffusion weighting. Images were analyzed by using in-house software written in Matlab (MathWorks, Natick, Mass). Images from all diffusion acquisitions were convolved with a Gaussian kernel with full width at half maximum (FWHM) of 3 mm within the plane to reduce noise level in accordance with standard diffusion image analysis procedures (24). Subsequently, the diffusivity maps of DWI were computed from conventional DWI by using a voxel-wise standard logarithmic algorithm (23). The diffusivity maps of monoexponential fitting approach, DKI, and SEM were computed from diffusion acquisition 1 by using a nonlinear fitting algorithm (15–17).

QSI analysis was performed voxel-wise by using fast Fourier transform to compute the displacement probability density function from diffusion acquisition 2 (19). Diffusion-weighted signal was mirrored around a q value of 0 cm^−1^ before fast Fourier transform (20). The FWHM was obtained from the resulting distribution, quantifying the extent of diffusion (25). To evaluate the dependence of QSI on sampling density, the analysis was repeated for a downsampled 11 q values from diffusion acquisition 2, linearly spaced from 30.1 to 655 cm^−1^.

Regions of interest were drawn by a single operator in MRIcron (University of South Carolina, Columbia, South Carolina, *https://people.cas.sc.edu/rorden/mricron/index.html*) on DWI diffusivity maps to delineate the entire tumor mass from surrounding breast tissue (26). Voxels within the tumor core from the diffusivity map from each diffusion method (FWHM map in the case of QSI) were subsequently extracted to form a histogram for each tumor. The median and skewness were then computed from the histogram distribution as the markers of average and asymmetry of histogram distribution, respectively, for the corresponding diffusion method (11,12,27).

### Histopathologic Analysis

Routine histologic assessment was performed to obtain the phenotype classification, tumor diameter, and grading on the basis of the Nottingham Grading System (28). From each tumor, a representative hematoxylin-eosin–stained section was digitally imaged at a pixel size of 0.505 μm on a slide scanner (Aperio CS2; Leica Biosystems, Melbourne, Australia). The regions of the images covering the tumor area were then fragmented into image tiles of 800 × 800 pixels (0.16 mm^2^; Fig E1 [supplement]). A total of 94 tiles among the overall 19 547 tiles from the participants were excluded because of inadequate staining quality or mounting artifact during slide scanning. Images were processed by using software (ImageJ; National Institutes of Health, Bethesda, Md) to obtain binary masks of stained nuclei material, with an intensity threshold applied to the red channel image (8,9,14). The cellularity of an image tile was computed as the ratio between the image mask positive for nuclei material and the tile size (8, 9, 21). Median and skewness values were extracted from cellularity histogram distributions compiled from all the image tiles within each tumor section.

### Statistical Analysis

Statistical analysis was performed by using software (SPSS version 24.0; IBM, Armonk, NY). All cohort distributions of skewness and median values were confirmed to be normally distributed by using the Shapiro-Wilk method. Note that this may be a result of underpowering because with a small sample virtually all distributions are indistinguishable from normal. Any overall differences in mean values of skewness between diffusion imaging methods was examined by using within-subjects analysis of variance. Post hoc paired *t* test comparison was used to investigate whether there was a significant difference between the skewness values obtained from QSI and other diffusion methods by applying Bonferroni correction for multiple comparisons for a significance level of *P* less than .017. To investigate whether QSI yields a higher relative effect gradient compared with existing diffusion methods, the skewness values from each diffusion method were correlated against the skewness values from QSI. The relative effect gradient was evaluated as the line of best fit gradient and reported as percentage increase. The correspondence of the median and skewness between diffusion imaging techniques and the underlying cellularity were examined by using Spearman correlation. In addition, to examine whether QSI analysis was feasible for a reduced sampling density, the correspondence between the skewness and median values obtained from QSI analysis at 11 and 32 q values was examined by using Pearson correlation.

## Results

Individual tumor characteristics of all 20 invasive carcinoma specimens including tumor type, size, and hormone receptor status are in Table 1.

**Figure 2:**
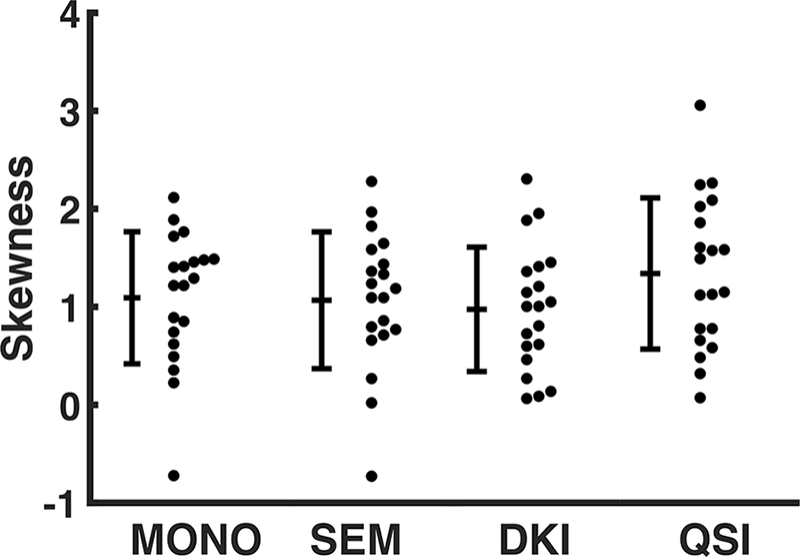
Plot shows comparison of skewness of diffusion-weighted imaging techniques. The skewness from diffusivity obtained from each of the diffusion-weighted imaging techniques (the monoexponential model [MONO], stretched exponential model [SEM],and diffusion kurtosis imaging [DKI]) is shown and compared with the skewness obtained from q-space imaging (QSI) full width at half maximum. Each dot represents a single tumor value and the error bar represents the cohort mean ± standard deviation.

**Figure 3:**
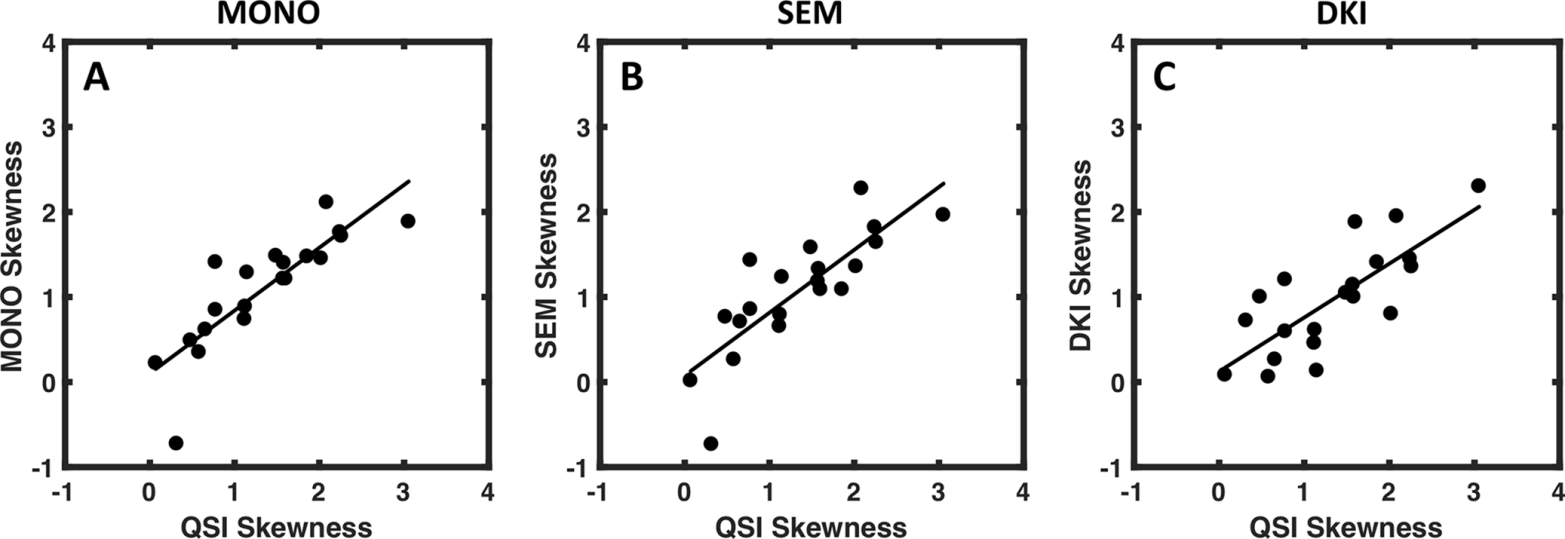
Graphs show correlation of skewness. The skewness of diffusivity obtained from, *A,* monoexponential model (MONO), *B,* stretched exponential model (SEM), and, *C,* diffusion kurtosis imaging (DKI) is plotted against the skewness obtained from q-space imaging (QSI) full width at half maximum. The line of best fit is shown, with each dot representing a single tumor value. The relative effect gradient was determined from the line of best fit gradient for skewness values. QSI yielded a higher relative effect gradient (percentage increase) compared with MONO (0.26 of 0.75; 35.1%), SEM (0.26 of 0.75; 35.1%), and DKI (0.37 of 0.63; 58.7%).

There was significant difference in skewness obtained from diffusion methods (*F* = 4.803; *P* = .015). The skewness from QSI (cohort mean, 1.34 ± 0.77 [standard deviation]) was significantly higher (*P* < .017) compared with the skewness from monoexponential fitting approach (1.09 ± 0.67; *P* = .015), SEM (1.07 ± 0.70; *P* = .014), and DKI (0.97 ± 0.63; *P* = .004) (Table 2, Fig 2). There was significant (*P* <.0005) linear correlation between the skewness from QSI and other diffusion methods, with QSI yielding a higher relative effect gradient (percentage increase) compared with monoexponential fitting approach, 0.26 of 0.75 (35.1%); SEM, 0.26 of 0.75 (35.1%); and DKI, 0.37 of 0.63 (58.7%) (Table 2, Fig 3).

**Table 2:**
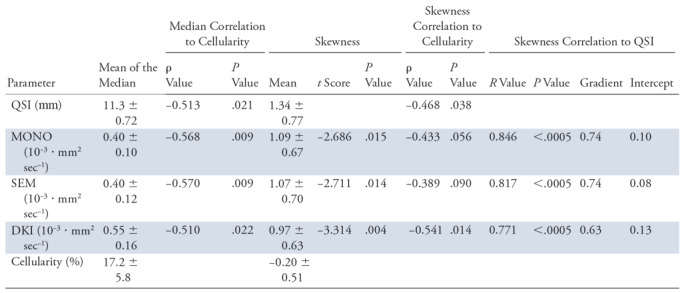
Comparison of Diffusion-weighted Imaging Techniques

Note.—Mean data are ± standard deviation. For each diffusion-weighted imaging technique, the cohort mean of the median and skewness of diffusivity is shown for the monoexponential model (MONO), stretched exponential model (SEM) and diffusion kurtosis imaging (DKI), and full width at half maximum for q-space imaging (QSI). The cohort mean of the median and skewness of cellularity is shown. The skewness obtained from each method compared against the skewness from QSI by paired sample *t* test is shown, with associated *t* score (*t*) and *P* value. The skewness obtained from each method compared against the skewness from QSI by Pearson correlation is shown, with associated Pearson correlation coefficient (*R*), *P* value, along with the gradient (relative measurement effect gradient) and intercept of the line of best fit. The median and skewness obtained from each diffusion method compared against that from cellularity by Spearman correlation is shown, with associated Spearman correlation coefficient (ρ) and *P* value.

**Figure 4:**
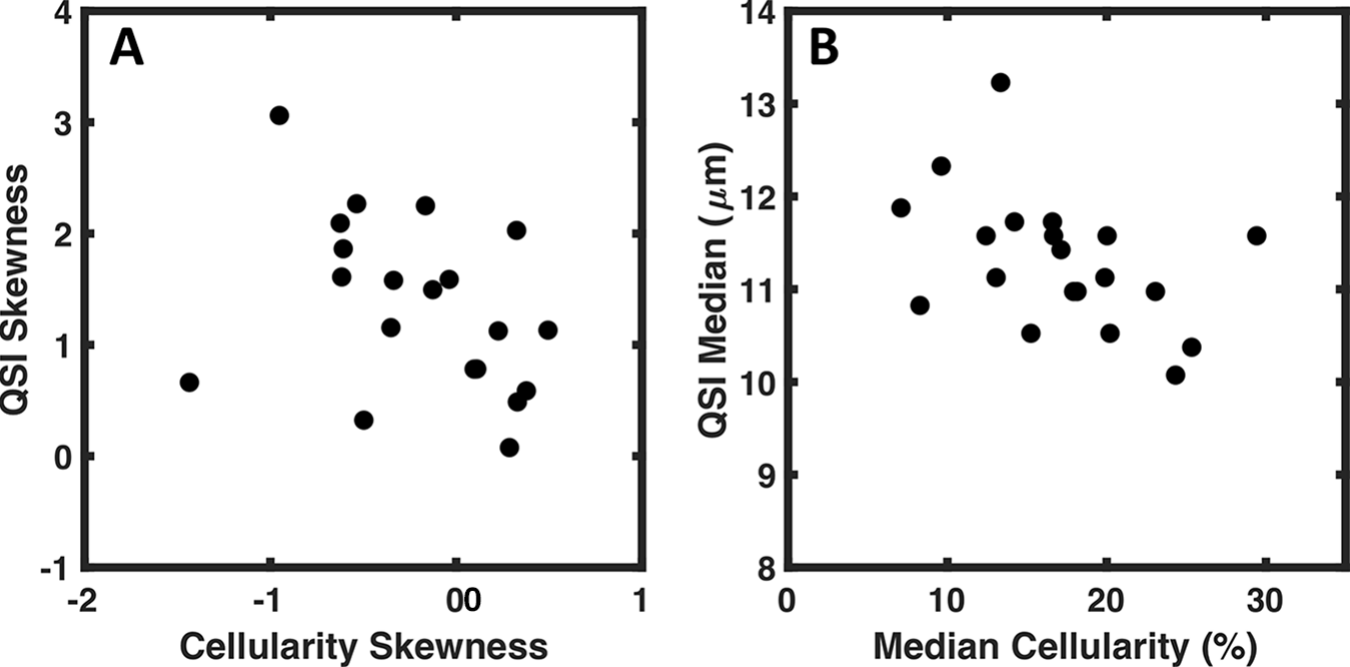
Scatterplot shows correlation between q-space imaging (QSI) and cellularity. The correspondence of the median and skewness between diffusion imaging techniques and the underlying cellularity were examined using Spearman correlation. *A,* Correlation between the skewness obtained from QSI plotted against the skewness of cellularity. *B,* Correlation between the medians from QSI and cellularity. Each dot represents a single tumor value.

There was a significant (*P* <.05) correlation between the skewness from cellularity and the skewness from QSI (ρ = −0.468; *P* = .038) and DKI (ρ = −0.541; *P* = .014) (Table 2, Fig 4). However, there was nonsignificant correlation between the skewness from cellularity and skewness from monoexponential fitting approach (ρ = −0.433; *P* = .056) and SEM (ρ = −0.389; *P* = .090). There was significant (*P* <.05) negative correlation between the median of cellularity and the medians from QSI, monoexponential fitting approach, SEM, and DKI (Table 2, Fig 4).

There was a significant correlation between the median and skewness from downsampled QSI and fully sampled QSI (Fig E2 [supplement]).

## Discussion

We found that the tumor cellularity obtained from QSI had an increased effect gradient compared with the other DWI techniques, providing a measurement with amplified skewness in the tumor histogram distribution and significant correlation to the cellularity skewness from histologic analysis.

The significantly higher values of skewness obtained from QSI corresponded to an increased measurement effect gradient and hence higher sensitivity to the underlying differences in breast tumors. With a positive response to treatment, there is a reduction in the amount of tumor cellularity, which corresponds to a reduced proportion of viable tumor tissue (6). The loss of cellularity is observed as a characteristic shift in the histogram distribution to higher diffusivity values and reduced skewness as the tumor composition becomes more homogeneous (11–13). Changes to skewness have been shown to precede changes to the texture and width of histograms in chemotherapy and radiation therapy treatment of advanced cervical cancers by using conventional DWI (29). Whereas previous studies have shown the added sensitivity of the use of skewness in detecting response at DWI (10) and dynamic contrast agent–enhanced imaging (30), the low percentage change in skewness relative to its high measurement error prevents identification of nonresponding participants (11–13,30). We find QSI to increase the effect gradient of skewness assessments, and therefore potentially it provides a marker with improved sensitivity in detecting the changes to tumor cellularity that occur with a positive early response to treatment.

Our results show QSI to provide sensitive noninvasive assessment of breast tumor cellularity, in agreement with results from in vivo imaging of meningioma (22) and preclinical imaging of excised esophageal carcinoma samples (21). Previous studies have demonstrated an inverse correlation between the cellularity of breast tumors and the average diffusivity obtained at conventional DWI (8,9), whereas others have shown nonsignificant correlation and limited specificity (31). In our study, we found a significant inverse correlation between the median cellularity and median values from all DWI techniques that is consistent with a previously reported pooled strength of association (ρ = −0.48) from conventional diffusion imaging of breast cancer (32). Tumor heterogeneity was accounted for by taking the average as the median value from the corresponding histogram distributions (33). A comprehensive assessment of average tumor cellularity was obtained from histologic analysis of whole slide sections. Histogram distributions of cellularity did not distinguish between malignant and nonmalignant cells. Although not investigated in this study, a large amount of tumor-infiltrating immune cells may influence the associations measured between diffusion properties and cellularity.

The skewness from QSI and DKI, compared with monoexponential fitting approach and SEM, showed significant inverse correlation with the skewness in cellularity, with QSI providing both fidelity to the underlying tumor histologic structure and the highest relative effect gradient. Therefore, QSI is a promising approach to noninvasively monitor changes in cellularity occurring with positive treatment response, where core biopsy can only provide partial sampling of cellularity and is incapable of assessing the whole tumor (34).

This study addresses the urgent clinical need to amplify the sensitivity of noninvasive cellularity markers through comprehensively evaluating QSI translated for clinical research on a 3.0-T MRI unit. This study investigated the sensitivity of QSI compared with existing radiologic methods in 20 large tumors of invasive carcinoma. Tumor cellularity was obtained from imaging whole-breast tumors freshly excised from participants and before routine histologic analysis, where previous studies have been limited to imaging smaller sections of tumor tissue that are surplus to the amount of tumor tissue required for histologic reporting (18,21). We demonstrate the pertinence of QSI to provide sensitive assessment of tumor cellularity and to be feasible by using a downsampled number of diffusion weightings for clinical breast imaging durations.

Our study had limitations. Because this study was limited to a cohort of 20 participants, narrow inclusion criteria were imposed on phenotype and tumor size to ensure statistical power for the comparison of effect gradient at an effect size clinically relevant for personalized care. The study was limited to grade II or III invasive carcinomas with large tumor size, excluding lobular type and grade I tumors because invasive ductal carcinomas are the most commonly diagnosed type of breast cancer (35). To meet the clinical requirements of routine histologic analysis following the imaging study, buffered 10% formalin solution was added to the freshly excised breast tissue before imaging overnight on the same day as excision. Although tissue fixation affects diffusion measurements, tumors were imaged before fixation, with formalin penetration into tissue typically at 1 mm per hour followed by an additional 24 hours for the tumor to become fixed (36,37), allowing whole tumors to be imaged at the point of excision rather than cut sections of tissue surplus to routine histologic analysis. Tumors were further monitored for experimental stability by an additional three repeated DWI acquisitions to encompass the diffusion acquisitions, and no significant difference in median or skewness from diffusivity was found (data not shown). A 32-channel head receiver coil was used so that excised tumors could be placed at the isocenter of the imager to allow power calibration and shimming functions by using a clinical MRI unit. Translation of QSI into the clinic requires optimization to ensure adequate signal-to-noise ratio, through adjustment of voxel size, imaging duration, and receiver coil choice.

Future in vivo studies should investigate the reproducibility of QSI markers and association to a wider range of tumor sites, phenotypes, and treatments. The inclusion of additional markers of histogram shape and texture may also offer complementary information to radiomics analysis (38), diagnostic discrimination of lesion malignancy (39), and existing formal histopathologic classifications (34) to include clinically relevant information for the pretreatment tumor status and extent of residual disease.

In conclusion, QSI had increased effect gradient compared with monoexponential fitting approach, SEM, and DKI diffusion imaging techniques for evaluating whole-breast tumor cellularity, and yielded fidelity to the underlying tumor histologic structure. QSI provides a promising noninvasive approach to elucidate cellularity in whole-breast tumors at 3.0 T.

## SUPPLEMENTAL FIGURES

Figure E1:

Figure E2:
