# Supplementary figures and images for: q-Space Imaging Yields a Higher Effect Gradient to Assess Cellularity than Conventional Diffusion-weighted Imaging Methods at 3.0 T: A Pilot Study with Freshly Excised Whole-Breast Tumors
Source: Radiol Imaging Cancer. 2019 Sep 27;1(1):e190008. doi: 10.1148/rycan.2019190008 (PMC7983771; doi:10.1148/rycan.2019190008)

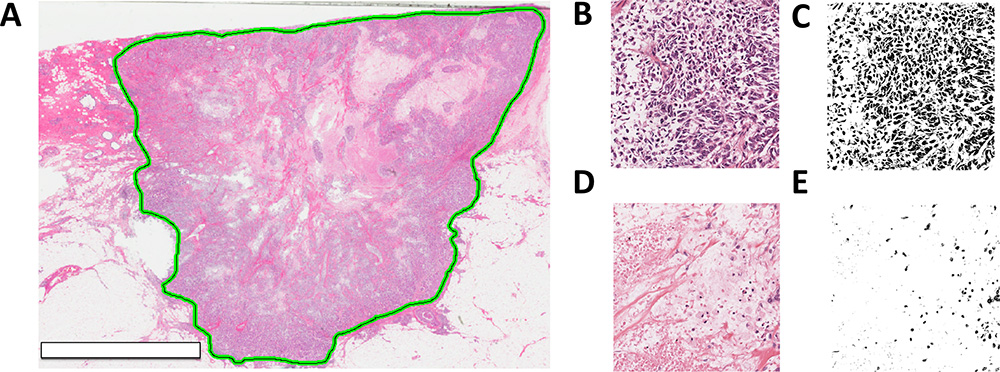

Supplement: Figure E1: [file rycan190008suppf1.jpg]

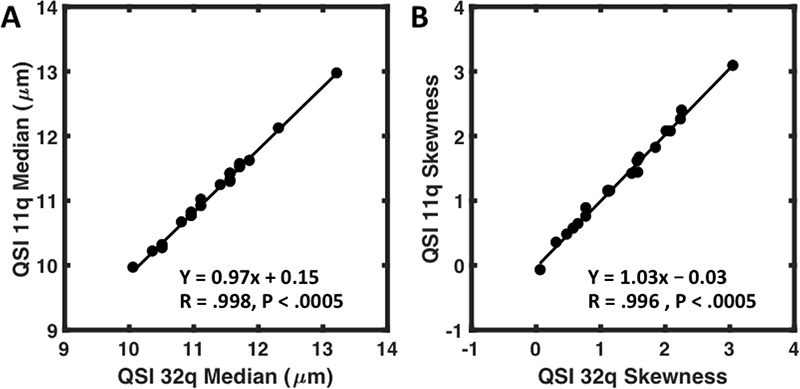

Supplement: Figure E2: [file rycan190008suppf2.jpg]
